# *Kluyveromyces marxianus* KLU1 Increases the Cell Density and Survival of *Lacticaseibacillus rhamnosus* GG in Dried Kurut

**DOI:** 10.1002/mnfr.70460

**Published:** 2026-04-09

**Authors:** Aigerim Tuganbay, Amin Yousefvand, Anna Kolsi, Dongming Zhang, Per Erik Joakim Saris

**Affiliations:** ^1^ Department of Microbiology Faculty of Agriculture and Forestry University of Helsinki Helsinki Finland; ^2^ Helsinki Institute of Sustainability Science (HELSUS) University of Helsinki Helsinki Finland; ^3^ Oy Medfiles Ltd Vantaa Finland

**Keywords:** dried kurut, *Kluyveromyces marxianus*, *Lacticaseibacillus rhamnosus* GG (LGG), probiotic, viability

## Abstract

Kurut is a traditional acid‐coagulated dairy fermented product from Kazakhstan, China, Turkey, and some Central Asian countries. It can be consumed either fresh, semi‐dried, or dried. In this study, probiotic *Lacticaseibacillus rhamnosus* GG (LGG) dried kurut was produced with and without the addition of *Kluyveromyces marxianus* KLU1, a yeast strain, we earlier isolated from kurut. *K. marxianus* KLU1 increased the cell count of LGG, enabling a probiotic level of >10^6^ CFU/g for more than 100 days in dried kurut stored at +4°C. Addition of LGG and *K. marxianus* KLU1 in kurut had no adverse effects on sensory attributes or physicochemical properties. *K. marxianus* KLU1 may also be useful in other applications where the aim is to use in situ approaches to increase the cell number of added probiotic *lactobacilli* in food fermentations.

## Abstract 2

A two‑step fermentation process for dried fermented milk balls was developed using *Lacticaseibacillus rhamnosus* GG alone or in combination with *Kluyveromyces marxianus* KLU1 or kefir grains; the yeast’s identity and phylogenetic position were confirmed by ITS (ITS1–ITS4) sequencing, and storage stability was evaluated by monitoring pH and *L. rhamnosus* GG viability, showing that co‑fermentation with the yeast better preserved acidity and probiotic counts during long‑term refrigerated storage.

**Abbreviations**
AOAC: Association of Official Analytical Chemists
ATCC: American Type Culture Collection
aw: Water activity
BLAST: Basic Local Alignment Search Tool
CFU: Colony‐forming units
HMW: High molecular weight
ITS: Internal transcribed spacer
KLU1: *Kluyveromyces marxianus* isolate 1
LGG: *Lacticaseibacillus rhamnosus* GG
MEGA: Molecular Evolutionary Genetics Analysis (software)
NCBI: National Center for Biotechnology Information
PBS: Phosphate‐buffered saline
ROS: Reactive oxygen species
SDA: Sabouraud Dextrose Agar
SPSS: Statistical Package for the Social Sciences
UHT: Ultra‐high temperature

## 1 Introduction

Fermented milk products have been produced for centuries, as fermentation is ancient and effective method for milk preservation. One such products is kurut (kurt, kurut, kaskh; in Kazakh and Russian, ‘құрт’), a sun‐dried traditional fermented food [1] that can be stored for several years without deterioration [2]. Kurut is produced in Middle Eastern and Central Asian countries, and in some provinces of China. Different variations in the name and pronunciation of kurut can be found among the nationalities in these regions, even when the product is produced using almost the same technology [3]. Throughout the centuries, traditional kurut has maintained its significance and has historically been made using milk from various animal species, including sheep, goats, cows, and even mares and camels in some regions of Kazakhstan [4]. In modern times, kurut has gained popularity in Kazakhstan with the addition of various ingredients such as herbs, plant extracts, dried fruits, and nuts, as well as being smoked. Several traditional techniques exist for its preparation, depending on the form—semi‐solid or dried. To prepare dry kurut, fermented milk is put into a pot and continuously cooked with frequent stirring until a significant amount of liquid has evaporated. The resulting mixture is then placed in a linen cloth bag and hung in the shade for several days to let excess moisture drain away. The collected coagulated milk proteins are then mixed with salt or butter, and hand‐shaped into balls. These balls are air‐dried in the shade or exposed to sunlight and wind for a few days, becoming harder as they dry [5]. The size, weight, and shape of these balls can vary depending on the region and country, ranging from flattened to oval, round, or conical forms, with diameters ranging from around 0.5 cm to 5 cm. The flavor of kurut is generally dry and salty, although it may have some sweet and sour notes.

In a previous study, semi‐solid kurut with the inclusion of *Lacticaseibacillus rhamnosus* GG (LGG), an extensively studied probiotic bacteria [6] and a kurut yeast isolate [7]. Briefly, the kurut was prepared via a two‐step fermentation: UHT milk (1.5% fat) with 3% glucose was inoculated with 1% LGG and incubated at 37°C for 19 h, followed by the addition of the yeast isolate (1%) and further incubation at 37°C for 24 h.

Probiotics are defined as live microorganisms that confer a health benefit to the host when administered in adequate amounts [8]. In the context of probiotics, their viability is the key requirement for producing probiotic containing foods [9]. This factor is critical because probiotic bacteria must not only exhibit health benefits, but also remain viable at the beneficial minimum therapeutic level (from 10^6^ to 10^8^ CFU/mL) throughout the product's shelf life [10]. Many factors affect the viability and stability of probiotics in dairy products. These include TTA (titratable acidity), potential of hydrogen (pH), homogenization, dissolved oxygen content, H_2_O_2_, storage temperature, type of probiotic, lactic acid concentration, and species and strains of the associative organism [11]. In this context, there are studies exploring how to increase probiotic viability with microencapsulation, and prebiotic addition techniques have been developed [11]. In previous study semi‐solid kurut, LGG maintained viable counts above10^7^ CFU/g throughout 22 days of storage at 4°C. LGG populations increased during the first 5 days of storage, reaching approximately 8.5 log CFU/g, followed by a gradual decline to ∼7.5 log CFU/g by day 22. Importantly, the presence of the kurut yeast isolate did not adversely affect probiotic survival. The yeast isolate used in that study was initially identified as *Cryptococcus laurentii* based on phenotypic characterization; however, subsequent ITS sequencing in the present study demonstrated that the strain belongs to *Kluyveromyces marxianus*.

The major feature of *K. marxianus* is the ability to utilize lactose as a carbon source [12]. This may explain the frequent isolation of *K. marxianus* from dairy sources such as fermented milks, yoghurt, and cheese [13]. Moreover, this yeast exhibits a fast growth rate, thermotolerance (up to 52°C) [14], high secretory capacity, and the ability to produce ethanol by fermentation. This makes *K. marxianus* a promising candidate for various biotechnological processes and industrial applications [15].

The objectives of this study were to develop probiotic dried kurut containing both *L. rhamnosus* GG and *K. marxianus*, investigate the viability of LGG in the kurut, and to evaluate if LGG and *K. marxianus* as starters in kurut production affected sensory attributes or physicochemical properties.

## 2 Experimental Section

### 2.1 DNA Isolation From Yeast for Molecular Identification

For molecular identification, DNA extraction of *Cryptococcus laurentii* [7] was performed by combining the method described by Chen et al. (2020) [16] with the Monarch HMW DNA Extraction Kit for Tissue. Log‐phase yeast cells (15 mL) were harvested through centrifugation at 5000 × *g* at 16°C for 15 min and washed twice with phosphate buffer saline (PBS). After washing, the cells were suspended in 1 mL of cold PBS, frozen at −18°C, and allowed to thaw at room temperature. The thawed sample was homogenized by vortexing, and this freeze–thawing cycle was repeated twice.

The resulting pellet was suspended in 400 µL of spheroplast buffer (1 M sorbitol, 1 × PBS pH 7.4, 0.1 M EDTA) containing 10 µL of zymolyase (5U/µL). The sample was then incubated at 30°C for 20 min. Spheroplasts were pelleted by centrifugation for 3 min at 300 × *g*, and the supernatant was discarded. Spheroplast pellets were resuspended in 150 µL of cold 0.5 M NaCl.

For further processing, 450 µL of HMW gDNA Tissue Lysis Buffer (New England Biolabs T3061L) mixed with 15 µL of Proteinase K was added to the tube. The mixture was incubated at 56°C for 30 min. Subsequently, 10 µL of RNase (10 mg/mL) was added, and the tube was incubated at 37°C for 10 min. To this, 50 µL of 5 M NaCl and 100 µL of 10% SDS were added, followed by vigorous manual shaking. The freeze–thawing cycle was repeated.

For separation, 0.5 volume of a tissue separation buffer (New England Biolabs T3062L) was vigorously mixed into the solution, followed by centrifugation at 10 000 × *g* for 10 min. The supernatant was collected for further processing. An equal volume of chloroform‐isoamyl alcohol (24:1) was mixed with the supernatant and centrifuged at 10 000 × *g* for 10 min. The DNA was precipitated by adding two volumes of absolute cold ethanol and 0.1 volume of 3 M sodium acetate to the supernatant. The sample was centrifuged at 10 000 × *g* for 2 min to collect the DNA pellet, which was air‐dried and dissolved in elution buffer (10 mM Tris, pH 9.0, 0.5 mM EDTA).

### 2.2 PCR Conditions

The *Cryptococcus laurentii* strain [7] was originally identified by strain‐specific carbon compound assimilation pattern using the yeast identification system API 20C AUX (BioMerieux, France). For more exact taxonomic identification of the *Cryptococcus laurentii* strain, its ITS region was amplified for sequencing. One µL of the target DNA solution was amplified in a 50 µL reaction volume containing 10 µL of 5× Phusion HF Buffer*, 1 µL of 10 mM dNTP mix (Thermo Fisher Scientific), and 2.5 µL each of the forward ITS1 (5’‐TCCGTAGGTGAACCTGCGG‐3’) and reverse ITS4 (5’‐TCCTCCGCTTATTGATATGC‐3’) primer, as described by [17]. In addition, 0.5 µL of phusion DNA polymerase and 32.5 µL of nuclease‐free water were added. The PCR program consisted of the following steps: initial denaturation at 98°C for 30 s, 30 cycles of denaturation at 98°C for 10 s, annealing at 63°C for 10 s and extension at 72°C for 30 s, and a final extension 1 cycle at 72°C for 5 min. The amplification reaction was performed with a SimplyAmp Thermal Cycler PCR System. Extracted DNA was stored at −20°C and sent for sequencing.

### 2.3 DNA Sequencing and Identification

Sequencing was performed on the Illumina MiSeq platform in the DNA Sequencing and Genomics Laboratory, Institute of Biotechnology, University of Helsinki. Forward and reverse ITS reads were edited, reverse‐complemented, and assembled into a consensus contig using MEGA V11 [18]. The consensus sequence was compared against the NCBI GenBank nucleotide database using the BLASTn algorithm [19]. Identification to the species level was based on ≥98% sequence identity and ≥95% query coverage with reference sequences. The top BLAST hit corresponded to *Kluyveromyces marxianus* and the strain was named KLU1. The ITS sequence generated in this study has been deposited in GenBank under accession number PX117415, and a phylogenetic dendrogram was built (Figure 2). Evolutionary analysis was conducted using Neighbor‐Joining and Bootstrap methods.

### 2.4 Preparation of Kefir Grains Starter as a Control Group

Kefir grains were bought from a prebiotic company called “Prebiotic Ltd.” in Tehran, Iran, and were stored at −18°C. To prepare kefir, the grains were placed in ultra‐high temperature (UHT) milk (Milbona) with 1.5% fat obtained from LIDL (Helsinki, Finland) and kept at room temperature for approximately 10 h. To keep the grains healthy, fresh milk was added every day at the same time. The grains were activated to increase the amount of kefir grain biomass, which was used to ferment milk for kurut production.

### 2.5 Starter Cultures

**FIGURE 1:**
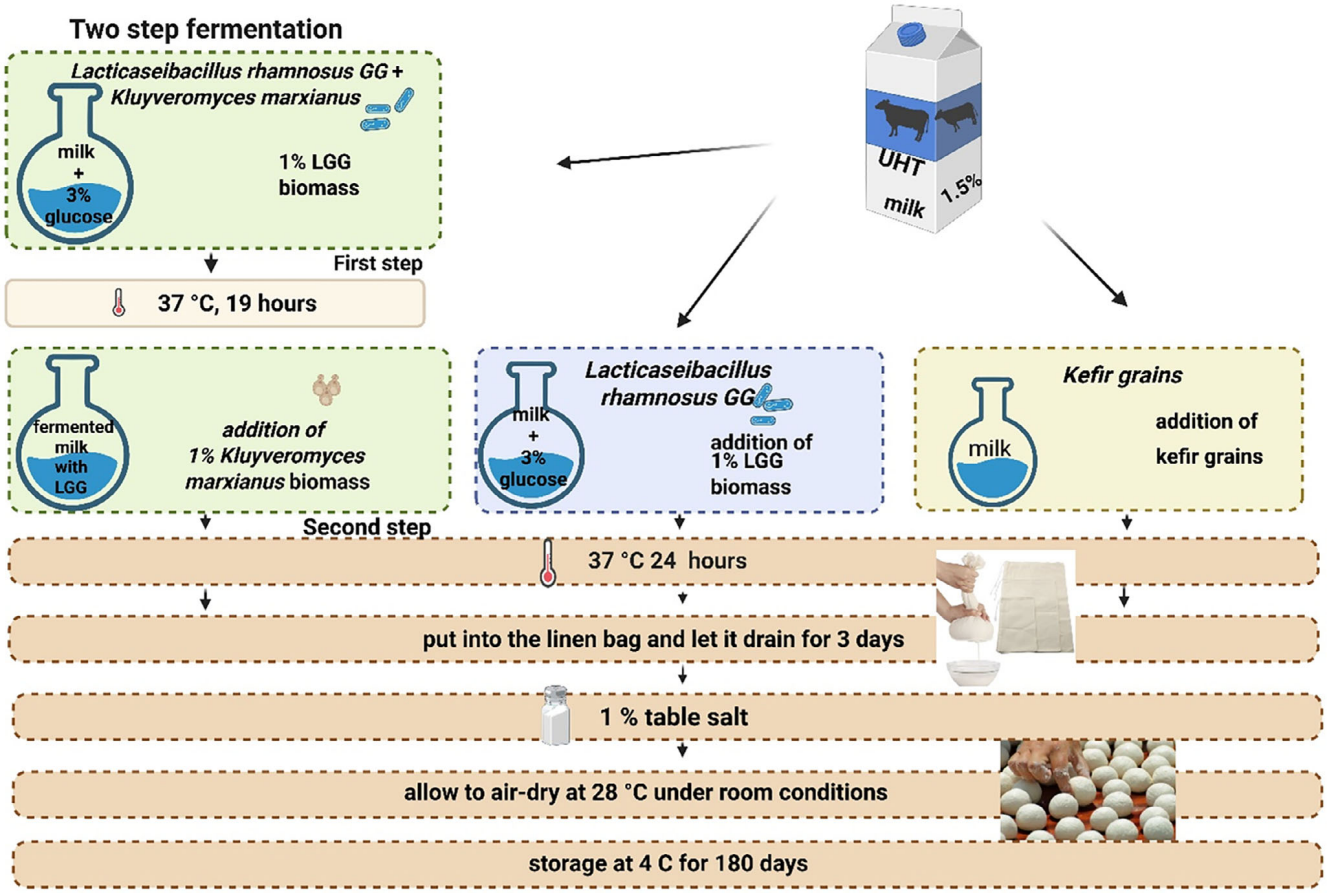
Manufacturing flowchart of different formulations of kurut. Control: Kurut produced with kefir grains; LGG‐Cont: Kurut containing LGG; LGG‐KM: Kurut containing LGG and *Kluyveromyces marxianus* KLU1; LGG: *Lacticaseibacillus rhamnosus* GG.

*Lacticaseibacillus rhamnosus* GG (LGG; ATCC53103) was activated in MRS (DeMan, Rogosa, and Sharpe) broth and cultured at 37°C for 24 h under anaerobic conditions. Anaerobic condition for the growth of LGG was done using anaerobic jar with Anaerocult A (Merck, Millipore) according to the manufacturer's instructions. The yeast *Kluyveromyces marxianus* KLU1 was activated in YPD broth and cultured at 37°C for 24 h, a temperature within optimal growth range reported for this thermotolerant dairy yeast [20] After incubation, 50 µL of LGG and *K. marxianus* KLU1 strains were sub‐cultured in plastic tubes containing 50 mL of MRS and YPD broth, respectively. LGG was cultivated anaerobically at 37°C for 24 h, whereas yeast cultures were incubated aerobically at 37°C for 24 h. The cells were harvested by centrifugation (4000 × *g*,10 min, 20°C), was washed twice with Ringer's solution and resuspended in 10 mL of UHT milk. Cell suspensions were adjusted to a final concentration of 1 × 10^8^ CFU/mL. Standardized bacterial and yeast suspensions were inoculated into milk at 1% (v/v) each. Fermentation was performed using two‐step process, consisting of a primary fermentation followed by a secondary fermentation stage, as illustrated in Figure 1.

### 2.6 Manufacturing of Kurut Samples

The kurut preparation was performed as described previously [7], including the addition of glucose to the milk prior to fermentation. The semi‐solid kurut preparation was finished by adding 1% (w/w) salt to the drained product and making the rolls by hand at 28°C, which were then left to dry for 3 days. Next, the kurut samples were cooled and stored at 4°C. During storage, the probiotic LGG and yeast viability were monitored for up to 180 days at 4°C, whereas physicochemical properties on days 1, 5, 10, 15, and 22 days of storage only. The process of making kurut is illustrated in Figure 1, created with BioRender. Created in BioRender. Tuganbay, A. (2025).

### 2.7 Physicochemical Analysis of Kurut

For the determination of pH and total titratable acidity, kurut samples were homogenized with distilled water (1:10, w/v). The pH values of the kurut were determined using a pH‐meter electrode (Thermo Orion Model‐420A). In addition, total titratable acidity (TTA) was measured using the AOAC official method as per standard procedures [21].

The moisture content of the kurut samples was measured according to the AOAC official method (AOAC, 1995). Each kurut product (5 g) was placed in an oven at 105°C for 3 h. Next, a reading was taken at a constant weight. The moisture content was then expressed as the percentage (%) of the dry weight of the sample. Based on the weight of the residue obtained from the moisture content analysis, the total solids of each of kurut sample were measured and expressed as the percentage (%) according to AOAC (AOAC, 2006). The ash content of each of kurut sample was measured at 550°C according to AOAC (AOAC, 1995) and expressed as the inorganic residue left as a percentage of the total weight of incinerated kurut. The water activity of kurut samples was measured at 25°C using an a_w_‐meter (Novasina LabMaster, Novasina, Switzerland).

### 2.8 Total Count and Viability of *Lacticaseibacillus rhamnosus* GG in Kurut

To access the viability of LGG and yeast, microbial populations in kurut samples were monitored during storage at 4°C and expressed as log colony‐forming units (CFU) per gram of the product (log CFU/g). First, 1 g of each sample was transferred to 9 mL of physiological saline solution and homogenized using a vortex mixer for 30 s. Samples were then serially diluted using the spread‐plate technique in MRS medium supplemented with 0.01% of cycloheximide. The plates were then incubated at 37°C for 24–48 h in an anaerobic jar, where anaerobic conditions were generated using Anaerocult A (Merck, Darmstadt, Germany). Yeast counts were determined on Sabourad Dextrose Agar (SDA) supplemented with 0.01% (w/v) chloramphenicol and incubated aerobically at 30°C for 48 h. Total microbial count was determined on MRS agar without supplements following aerobic incubation.

### 2.9 PCR Conditions for Identification of LGG Colonies

To verify LGG colonies (Woodman, 2008), 20 colonies/plate were amplified in a 50 µL reaction volume containing: 20 µL of 10 × dream Taq Buffer*, dNTP mix (2 mM each; Thermo Fisher Scientific), 25 µL each of the forward (5’‐CGCCCTTAACAGCAGTCTTC‐3’) and reverse (5’‐GCCCTCCGTATGCTTAAACC‐3’) primers, described by Yousefvand et al. (2022Yousefvand et al. (2022), 2.5 µL of Dream Taq DNA polymerase, and nuclease‐free water to a volume of 50 µL. The PCR program consisted of the following steps: initial denaturation at 95°C for 3 min (1 cycle), 30 cycles of denaturation at 95°C for 30 s, annealing at 57.9°C for 30 s and extension at 72°C for 1 min, and a final single extension cycle at 72°C for 5 min. The amplification reaction was performed with a thermocycler (Mastercycler Gradient).

### 2.10 Sensory Analysis of Kurut

A sensory evaluation was conducted on the 11th day of storage at the University of Helsinki (Helsinki, Finland), following established sensory evaluation procedures for dairy products [22]. The evaluation was performed by a panel of 15 semi‐trained and experienced members, including students, academic staff, and faculty members. The kurut pieces were presented to the evaluators in 100 mL polyethylene cups, each bearing a unique 3‐digit random code. The evaluators assessed each sample individually and provided scores based on the parameters of flavor, body, texture, color, appearance, and overall acceptability. The 5‐point hedonic scale was used, with 1 representing dislike very much or unacceptable and 5 representing like very much or acceptable. Evaluators were instructed to rinse their mouths with drinking water before tasting each sample.

### 2.11 Statistical Analysis

All physicochemical measurements and microbial enumeration were carried out in triplicates. The data obtained for the physicochemical, and organoleptic evaluation of kurut samples were submitted to ANOVA and microbial populations (total counts, LGG viability, and yeast viability) were analyzed using a two‐way ANOVA of variance under the General Linear Model procedure and were then reported as mean ± standard deviations. Tukey's test was used to compare the means, and differences were considered as significant based on a *p* < 0.05, with higher levels of significance (*p* < 0.001) noted where applicable based on F‐test results. All statistical analyses were performed using SPSS (Version 27.0, IBM Corporation, New York, USA).

## 3 Results and Discussion

### 3.1 Molecular Identification of Yeast

**FIGURE 2:**
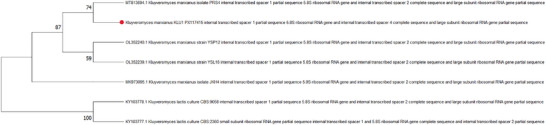
Phylogenetic dendrogram of the *Kluyveromyces marxianus* strains. Maximum Likelihood tree of ITS sequences showing the placement of isolate PX117415 (red circle) (= KLU1) within the *Kluyveromyces marxianus* clade.

Due to the inconsistent identification results from the API 20C system, we used ITS sequencing for accurate strain identification. The ITS1 and ITS4 Sanger reads were assembled into a 687‐bp contig (GC 43.2%) with no ambiguous bases. The forward and reverse reads overlapped by ∼619 bp with 99.3% pairwise identity, indicating high sequence quality. The sequences obtained from ITS regionwere then compared to strains available in the NCBI GenBank database (www.ncbi.nlm.nih.gov) using the BLAST tool. The results of the sequencing analysis confirmed that the strain was identified as *Kluyveromyces marxianus* and named KLU1. In detail, Figure 2 illustrates the phylogenetic relationships constructed using *K. lactis* as an outgroup to ensure accurate rooting. The analysis shows that *K. marxianus* KLU1 clustered significantly with *K. marxianus* isolate PRS4 (GenBank Accession MT813694.1) with 74% bootstrap support. A prior study found that yeast isolates obtained from traditional yogurt were identified as belonging to the *Kluyveromyces marxianus* [23].

### 3.2 Composition (Moisture, Ash, Total Solid, and Water Activity) of Dried Kurut Formulations

**FIGURE 3:**
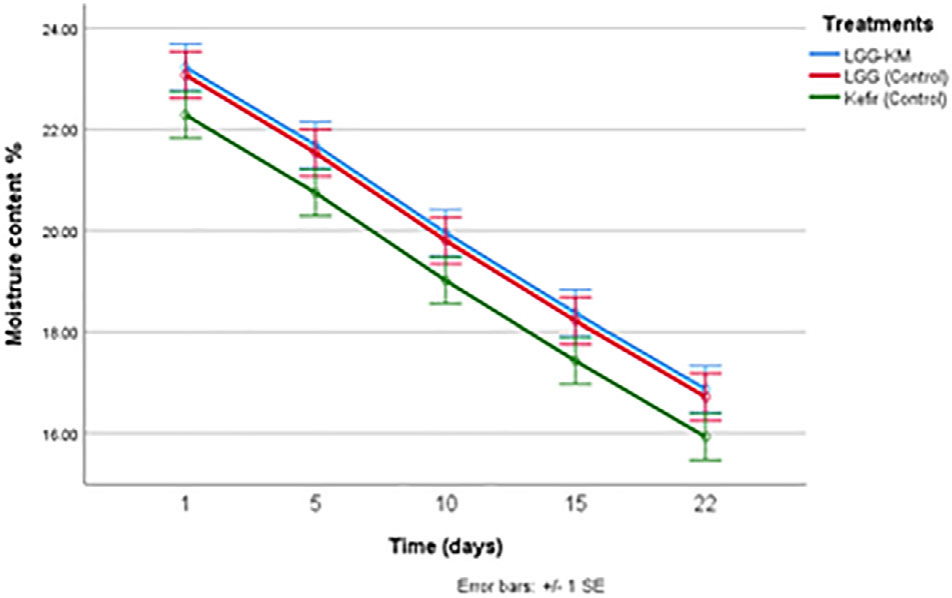
Moisture content (%) of dried kurut samples during 22 days of storage. Each point represents the mean of *n* measurements (*n* =3), and error bars indicate standard deviation.

One of the promising directions in the technology of preparation of fermented foods is the use of microorganisms in combined probiotic starters, which will not only expand the range of these food products, but may also improve their quality indicators and functional properties. In this study, dried kurut was made with kefir grains, (Kefir‐Control), kefir grains plus LGG (LGG‐Control) and kefir grains plus LGG and *K. marxianus* KLU1 (LGG‐KM). The moisture content of different kurut formulations is shown in Figure 3. The average moisture content of the samples with LGG‐KM, LGG‐Control, and Kefir‐Control groups were 20%, 19.9%, 19.1%, respectively. Our results were higher than the findings in the study by Kamber (2008) (12.1%), while they are lower than our previous results (27%–28.6%) [7, 24]. Such variations are likely attributable to differences in processing conditions, particularly drying intensity and duration, as well as product form. The lower moisture content reported by Kamber (2008) may reflect more intensive drying, whereas the higher values were observed previously consistent with semi‐solid nature of kurut and shorter moisture removal steps. In the present study, the intermediate moisture levels likely result from controlled drying and standardized storage conditions applied to dried kurut. The samples containing LGG‐KM and LGG‐control showed slightly higher values compared to the control group, although the differences were not significant (*p* > 0.05), and these moisture content values decreased throughout the storage days (*p* < 0.05).

**FIGURE 4:**
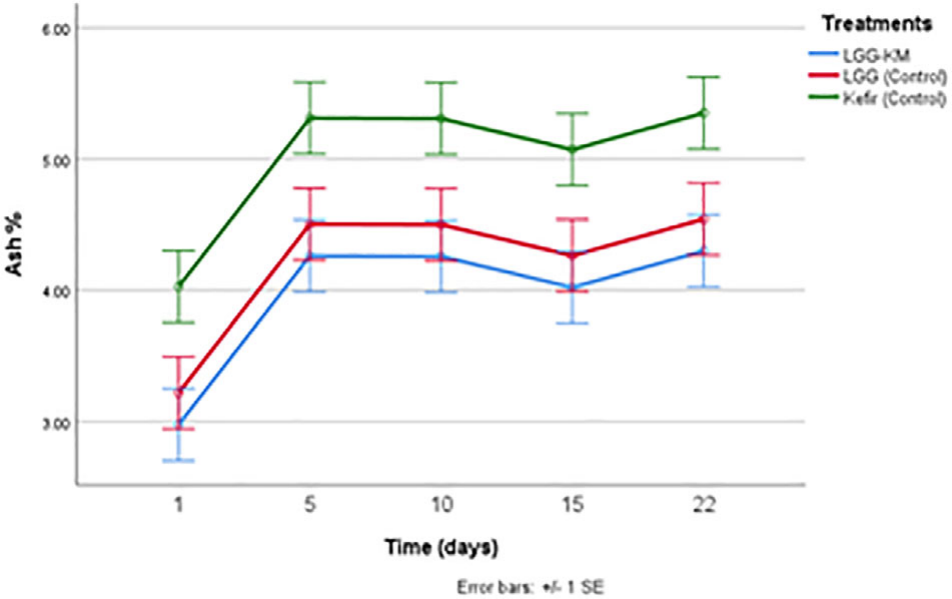
The ash content (%) of dried kurut samples during 22 days of storage. Each point represents the mean of *n* measurements (*n* =3), and error bars indicate standard deviation.

The results showed that the mean ash content of LGG‐KM, LGG‐Control and Kefir‐Control group samples were 3.95%, 4.25% and 5%, respectively (Figure 4). The kurut formulations in the control group showed remarkably higher ash content compared to the others (*p* < 0.05). These values were higher than those reported by Zhang et al. (2008), who showed that the ash content of kurut made from fermented yak milk samples varied between 0.8–0.95 (g/100 mL) [25]. However, these findings were similar to those reported by Waleed A Mustafa et al. (2013), who found that the ash amount was 3.77%–5.6% in white cheese samples [26]. Moreover, the amount of ash content was significantly enhanced with increasing shelf life (*p* < 0.05). This is probably because the moisture is removed from the kurut, and the surface of the kurut balls are covered with dry salt.

**FIGURE 5:**
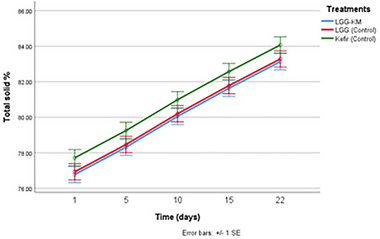
The total solid content (%) of dried kurut samples during 22 days of storage. Each point represents the mean of *n* measurements (*n* =3), and error bars indicate standard deviation.

Figure 5 shows the total solid content (%) of various kurut samples during storage. Samples in the Kefir‐Control group showed slightly higher total solid content compared to the other groups, but the difference was not significant (*p* > 0.05). It is noteworthy that there is a significant constant increase during storage, which is attributed to longer storage time; there is also a negative correlation between moisture content and total solid content.

**FIGURE 6:**
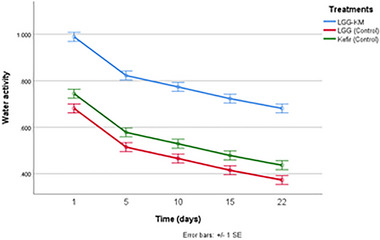
Water activity of dried kurut samples during 22 days of storage. Each point represents the mean of *n* measurements (*n* =3), and error bars indicate standard deviation.

Water activity (a_w_) is one of the most critical parameters in determining the quality and safety, and microbial stability of fermented foods, as it directly influences microbial metabolism, enzyme activity, and cell survival [27]. In this study, the water activity values of samples containing LGG‐KM were significantly higher compared to samples with LGG‐Control and Kefir‐Control group at all days of storage (*p* < 0.05). In general, water activity in the three groups decreased over time (Figure 6). Higher water activity levels have been reported to favor the survival of probiotic and starter cultures by maintaining cellular hydration and metabolic functionality, whereas reduced a_w_ values impose osmotic stress that can impair microbial viability during storage [28, 29, 30]. The relatively higher a_w_ observed in LGG‐KM samples may therefore have contributed to the improved survival of LGG and yeast during storage by providing a more favorable micronutrient for microbial persistence.

### 3.3 Physicochemical Properties of Kurut Samples

#### 3.3.1 pH and Total Titratable Acidity (TTA)

The most important indicator of dairy product freshness is acidity, which increases with the formation of lactic acid during the microbial life process. Active acidity is the concentration of hydrogen ions (N+), and its value is expressed as the hydrogen index pH, while titration acidity reflects the concentration of acidic components in dairy products.

**FIGURE 7:**
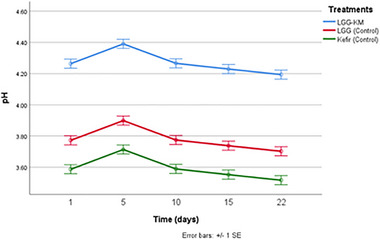
pH comparison of different kurut samples during 22 days of storage. Each point represents the mean of *n* measurements (*n* =3), and error bars indicate standard deviation.

The dynamics of pH changes during storage is illustrated in Figure 7. Dried kurut prepared using LGG‐KM treatment showed significantly higher pH than those made using other treatments (*p* < 0.05). In addition, the pH of the samples increased on day 5 of storage (*p* < 0.05) and decreased slightly toward the end of storage. In our previous study, we also observed a similar trend in semi‐solid samples of kurut prepared using starter culture with *L. rhamnosus* GG and yeast [7]. This phenomenon is likely due to the higher availability of nutrients produced by the yeast, which could enhance LAB metabolic activity and acid production, resulting in increased acidity and a corresponding decrease in pH.

**FIGURE 8:**
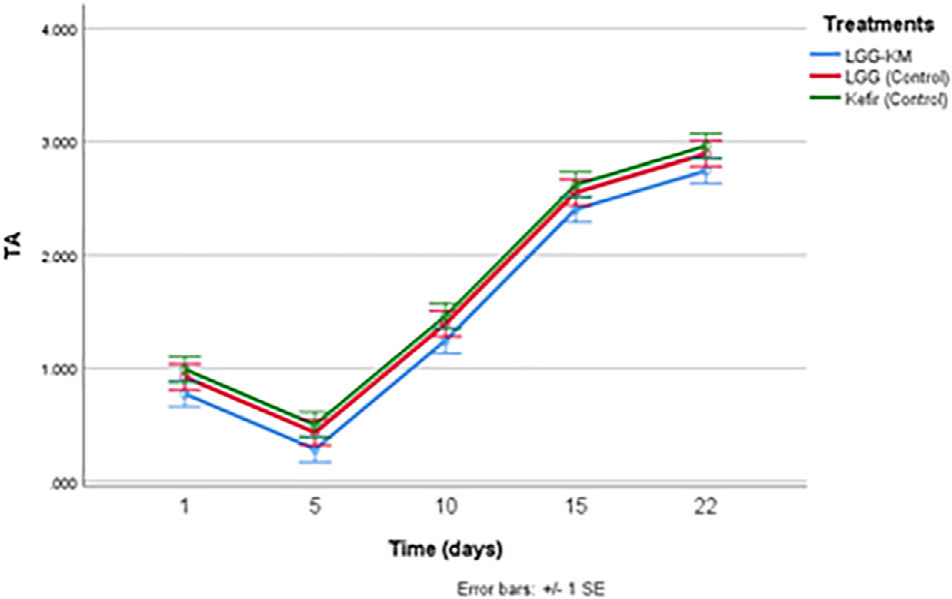
TTA comparison of different kurut samples during 22 days of storage. Each point represents the mean of *n* measurements (*n* =3), and error bars indicate standard deviation.

In contrast, TTA was lower in kurut samples treated with LGG‐KM compared to single‐bacteria fermentation or the control sample (Figure 8). However, no significant differences were observed between the different treatments (*p* > 0.05). Higher pH values and lower TTA in dairy products co‐cultivated with yeast compared to single LAB culture have been reported earlier [31]. For instance, Huang et al. (2020) also noticed that pH values decreased while titratable acidity increased during the fermentation of goat milk with LAB‐KM treatment. The increase in TTA observed during storage in all treatments reflects continued metabolic activity of probiotic LAB, primarily through the production of organic acids, such as lactic acid from residual fermentable substrates. This indicates that LGG remained metabolically active during the storage, contributing to gradual acid accumulation even under refrigerated conditions. Our outcomes displayed that TTA increased significantly from day 5 to day 22 of storage for all groups of treatment (*p* < 0.05).

### 3.4 Enhanced Microbial Viability in Kurut

The challenge for the food industry is to expand the production of probiotic products and enhance the probiotic properties of such compositions by utilizing other groups of probiotic microorganisms that can populate the gut and positively affect the host. One of the most important groups of probiotic organisms through which the properties of LAB‐based probiotic compositions can be improved is yeast. Much of the research on the production of co‐cultured yeast and LAB‐based products is devoted to the study of beverages [32, 33, 34, 35, 36, 37]. However, scientific and technical literature lacks data on dairy products. To the best of our knowledge, this is the first study to demonstrate the use of *K. marxianus* as a co‐starter culture with *L. rhamnosus* GG in dried kurut, resulting in enhanced probiotic survival during long‐term storage. Cell viability is essential for achieving the desired benefits of probiotics. In some cases, the shelf life of known probiotic compositions is significantly limited [38]. Herein, we used *K. marxianus* KLU1 and LGG as starter culture to produce kurut and analyze their viability during storage.

**FIGURE 9:**
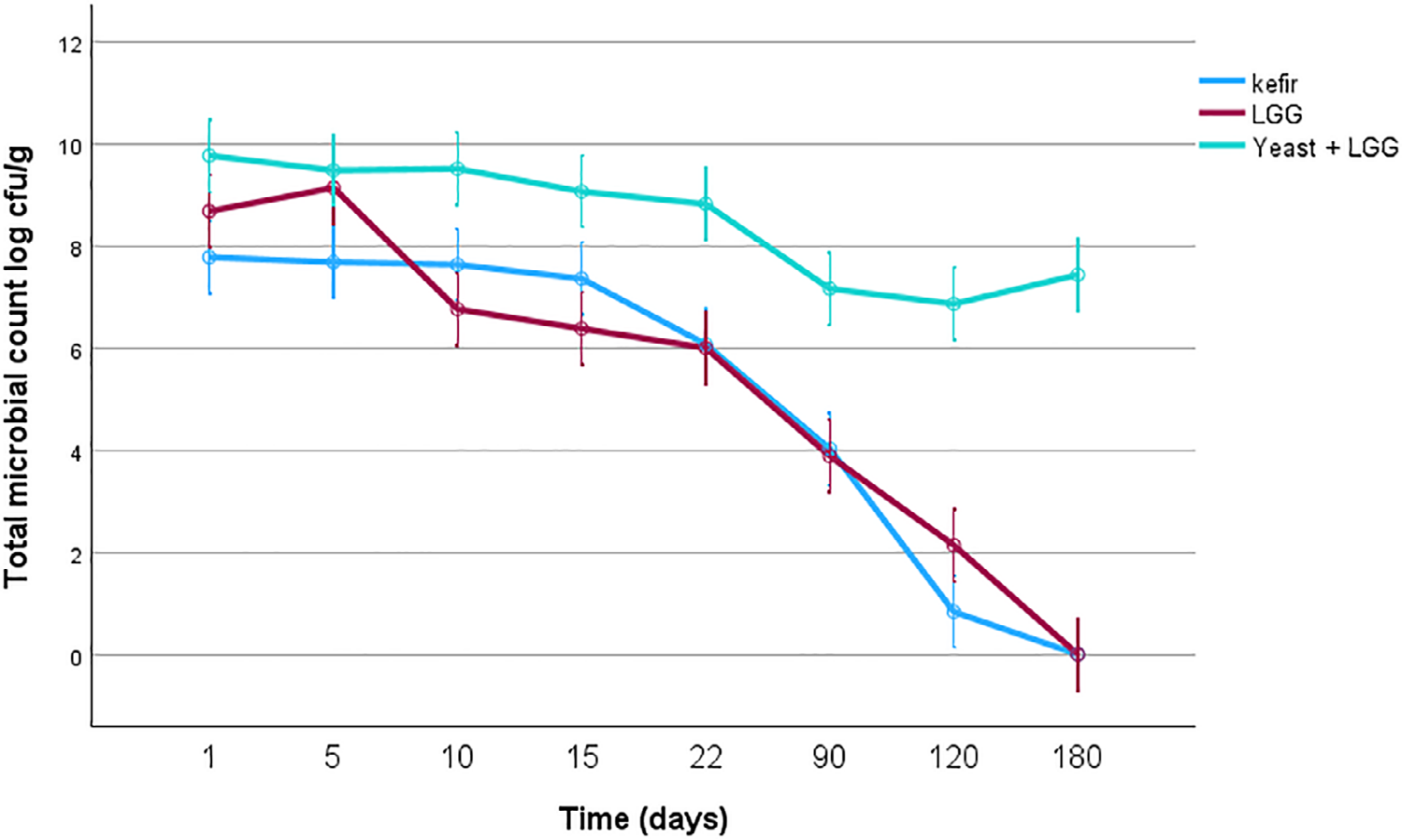
Total microbial counts in different kurut formulations during 180 days of storage. Each point represents the mean of *n* measurements (*n* =3), and error bars indicate standard deviation.

Total microbial counts (counted on MRS agar) in kefir, LGG, and LGG‐KM samples during storage are shown in Figure 9. Overall, LGG‐KM samples consistently exhibited higher (8.53 ± 0.3 log CFU/g) viable counts throughout storage compared to LGG alone and kefir samples declined rapidly after 22 days of storage to ∼6.0 log CFU/g, whereas no significant difference was observed between LGG and kefir formulations (*p >* 0.05). These results indicate that yeast co‐cultivation markedly enhances microbial stability in dried kurut.

Long term stability of LGG. The average number of viable LGG bacteria in LGG‐KM was 8.14 ± 0.3 log CFU/g at the end of storage time (22 days), while the LGG‐only group had already dropped to 6.01 ± 0.3 log CFU/g (Figure 11). For comparison, previous studies reported that the viability of encapsulated *L. rhamnosus GG* bacteria was around 7.7–7.9 log CFU mL^−1^ in buttermilk proteins [39]. In our previous study on semi‐solid kurut, LGG counts were maintained at approximately 7.0 log CFU/g after 22 days of storage [7]. Our current study shows an increase of about 1.5 log units in LGG viability when co‐cultivated with *K. marxianus* KLU1 in dried kurut, indicating improved probiotic survival despite the lower‐moisture matrix. During the first week of storage, LGG populations remained equivalent across both groups. However, the viability of LGG in LGG alone group begun a steady decline, reaching to 6.01 ± 0.3 log CFU/g by the 22^nd^ day of storage. In contrast, the yeast containing (LGG‐KM) group maintained significantly higher stability, staying above 8 log CFU/g until day 120 of storage at 4°C. Notably, the LGG counts in dried kurut with LGG‐KM was significantly greater than in other kurut formulations (*p* < 0.05).

Comparative profiles and synergistic mechanisms. While LGG remained stable in the co‐culture, yeast KLU1 followed a different profile. The comparative viability profiles of LGG and KLU1 are presented in Figure 12. Both strains exhibited similar initial counts (∼9 log CFU/g), but KLU1 declined rapidly after day 22, becoming undetectable by day 180. Interestingly, even as the yeast population declined, its initial presence provided lasting protection for the LAB. Goat milk fermented with *K. marxianus* and LAB showed significantly higher amounts of total LAB (log 9.65 CFU/mL) in the co‐fermentation system compared to single LAB fermentation [40] and Johansen et al. (2019) revealed that co‐culture of *K. marxianus* with *Lactococcus lactis* increased the viability of *L. lactis* [41]. The observed enhancement in probiotic stability is attributed to a multi‐faceted synergistic relationship between LGG and *K. marxianus* KLU1. It is hypothesized that the coexistence of LAB and yeast represents a mutually beneficial survival strategy, as LAB and yeast interactions in fermented foods have been shown to enhance microbial growth and persistence through nutrient cross‐feeding (e.g., amino acids and vitamins released by yeasts), modulation of redox conditions, and environmental conditioning of the fermentation matrix, thereby supporting the stability of both microbial groups [42, 43, 44]. This study on LAB and yeast interactions provides useful insight into the role of LAB in sustainably producing fermented products containing enough live probiotic cells. Moreover, *S. cerevisiae* was able to increase the survival of *L. rhamnosus* HN001, indicating that yeast metabolites play an essential role in enhancing LAB survival in fermented milk [45]. This might be due to changes in pH at the end of storage. Moreover, yeast plays a crucial role in fermentation, as it is involved in lactic acid and alcoholic fermentation. Toh and Liu (2017) demonstrated that yeast co‐cultivation reduced reactive oxygen species (ROS, one of the most critical barriers to LAB survival) levels in the culture medium, thereby creating a more reduced environment that supported the growth and survival of bifidobacteria [46]. Not only that, a study by Wang et al. (2023) showed that co‐cultivation of *Lactobacillus helveticus* SNA12 and *Kluveromyces marxianus* GY1 significantly increased the gastrointestinal tolerance under in‐vitro simulated gastric and intestinal conditions [47]. The authors attributed this effect to yeast‐LAB interactions, whereby the yeast provided protective metabolites and nutrients, enhanced stress resistance, and helped maintain cellular integrity of LAB cells under acidic and bile salt stress.

**FIGURE 10:**
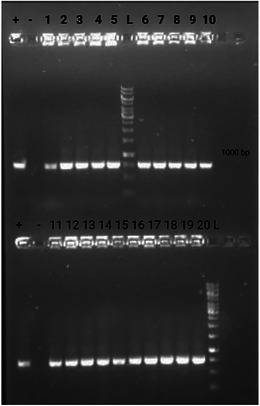
Agarose gel electrophoresis of PCR products from LGG colonies using specific primers. Lanes “+” and “‐” represent positive and negative controls, respectively; lanes from 1 to 20 represent individual colony isolates; lane “L” 1‐kb GeneRuler DNA ladder.

PCR amplification using LGG‐specific primers detected the expected 1200 bp fragment in all 20 samples (Figure 10). These samples corresponded to 20 randomly selected colonies recovered from MRS agar with cycloheximide plates used for LGG viability enumeration at the end of storage period (day 22). The 1% agarose gel electrophoresis confirmed the presence of single, distinct bands in these colonies, indicating successful identification of *Lactocaseibacillus rhamnosus* GG. Negative controls showed no amplification.

**FIGURE 11:**
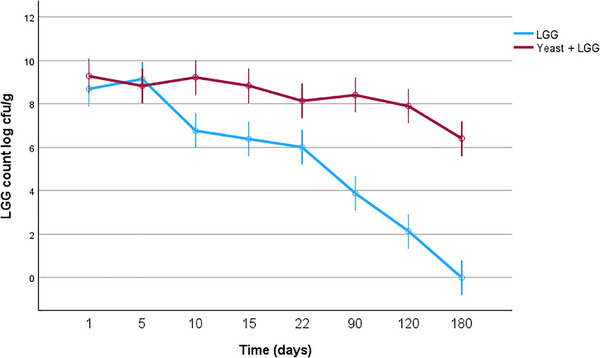
Viability of LGG in different formulations of kurut after 180 days of storage. Each point represents the mean of *n* measurements (*n* =3), and error bars indicate standard deviation.

In addition, Figure 11shows the amount of yeast on SDA. The samples with LGG‐KM had the highest (6.07±0.3 Log CFU/mL) yeast concentration. Differences in viable cell counts for all samples were significant (*p* < 0.05). Moreover, yeast plays a crucial role in fermentation, as it is involved in lactic acid and alcoholic fermentation. In yak kurut samples, the mean value of yeast was 8.33 ± 0.624 log CFU/mL [25]. Overall, both lactic acid bacteria and yeast contents in LGG‐KM samples were much higher than those in LGG and control (kefir) samples. We hypothesize that coexistence of LAB and yeast may be a survival strategy for mutual benefit, as it may promote intercellular interactions and enhance LAB and yeast growth. We believe that this study on LAB and yeast interactions provides useful insight into the role of LAB in sustainably producing fermented products containing enough live probiotic cells.

**FIGURE 12:**
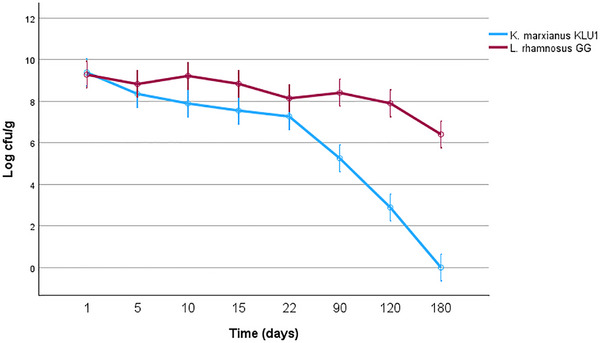
Total LGG and KLU1 counts in different formulations of kurut after 180 days of storage. Each point represents the mean of *n* measurements (*n* =3), and error bars indicate standard deviation.

### 3.5 Sensory Analysis

Using input from human sensory analyzers, organoleptics studies consumer's responses to various properties of food products, as well as food ingredients and intermediate forms of products. Sensory control allows the influence of all stages of food production promptly and purposefully. Subsequently, sensory analysis was performed to detect whether co‐fermentation of LGG and *K. marxianus* KLU1 would affect the organoleptic characteristics of kurut.

The results demonstrate that kurut formulations with LGG‐KM resulted in a significant change in flavor compared to the control samples (*p* <0.05) (Table 1). A similar result was found in an earlier study that showed how co‐fermentation of LAB and *K. marxianus* had a positive effect on the flavor of goat yoghurt [40]. This effect This effect is generally attributed to the formation of yeast‐derived aroma compounds, including esters, higher alcohols, aldehydes, and ketones, which contribute to fruity and complex flavor notes during LAB—yeast co‐fermentation (Huang et al., 2020). A study by Sun et al. (2023) showed that the flavor of milk co‐fermented with LAB and *K. marxianus* was significantly enhanced during storage due to the progressive accumulation of volatile aroma compounds [48]. However, LGG‐yeast treatment did not affect any other sensory properties (*p* > 0.05). In our previous study, we also observed insignificant differences in kurut formulations with LGG co‐cultured with yeast. The significant flavor improvement observed in the present study may therefore be related to the dried kurut matrix and processing conditions, which differ from the semi‐solid product and may intensify yeast‐mediated aroma development during storage. In addition, kurut samples with LGG‐KM treatment demonstrated a trend toward higher body and texture scores and overall acceptability, although they were not statistically significant (*p* > 0.05).

**TABLE 1:** Sensory evaluation of dried kurut formulations (*n* =15). Values are mean ± standard deviation.

Sensory attributes	Dried kurut formulations		
	LGG‐KM	LGG	Control
**Flavor**	3.47 ± 0.3^ab^	3.14 ± 0.1^a^	3.40 ± 0.2^a^
**Body and texture**	3.86 ± 0.2^a^	3.07 ± 0.2^a^	2.87 ± 0.2^a^
**Color and appearance**	3.29 ± 0.2^a^	3.50 ± 0.2^a^	3.33 ± 0.2^a^
**Overall acceptability**	3.64 ± 0.3^a^	3.35 ± 0.1^a^	3.23 ± 0.2^a^

^ab^ Different superscript letters within the same row indicate significant differences (*p* > 0.05).

## 4 Conclusion

Analysis of the data showed that combined LGG‐KM starter culture had sufficient biotechnological potential for use as components of combined starter cultures for kurut production. LGG gained a benefit in combined kurut LGG‐KM fermentation, as it reached significantly higher CFU/g compared to single strain inoculation. The higher cell concentration also led to a prolonged (> 100 days) survival of LGG over the threshold for a probiotic product. *Kluyveromyces marxianus* may be useful in other food fermentations when trying to reach high LGG counts by in situ growth during product fermentation.

## Ethics Statement

Ethical permission was not required. Participants were informed about the samples they would be evaluating and were made aware that the data collected would be used for research purposes as part of a research project. All participants voluntarily provided verbal consent to take part in the sensory evaluation sessions.

## Conflicts of Interest

The authors declare no conflicts of interest.

## Data Availability

The data that support the findings of this study are available from the corresponding author upon reasonable request.
